# Worse than the Disease? The Rash of *Lomatium Dissectum*

**Published:** 2018-05-18

**Authors:** Kenneth D. Marshall, Stephen L. Thornton

**Affiliations:** University of Kansas Health System, Department of Emergency Medicine

**Keywords:** lomatium dissectum, influenza, herb-drug interaction, traditional medicine

## Introduction

Influenza is a respiratory illness responsible for epidemic outbreaks of disease, and accounts for 500,000 to 1,000,000 emergency department (ED) visits in the United States each year.^1^ Because of the high prevalence of disease and its potential morbidity, emergency physicians must be facile in its diagnosis and treatment. In addition, some treatments for influenza, such as neuraminidase inhibitors, are associated with a higher risk of symptoms, such as gastrointestinal and central nervous system effects, which themselves can prompt ED visits.^2^ While front-line physicians have become adept at recognizing and managing both the symptoms of influenza and side effects of well-studied medical treatments, a further diagnostic challenge includes side effects of compounds or therapies recommended for influenza that are not well studied in Western medicine. Here, we present a report novel to the peer-reviewed medical literature of an adverse event due to the use of an herbal remedy for influenza.

## Case Report

**Figure 1:**
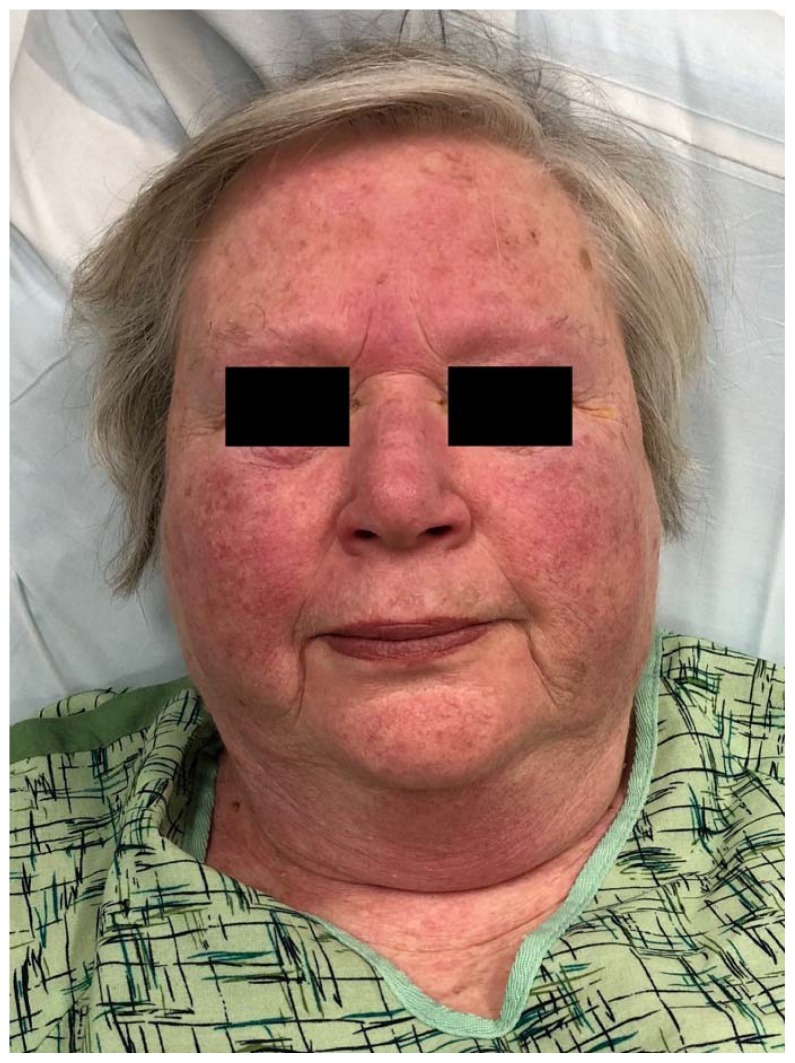
Facial rash.

**Figure 2:**
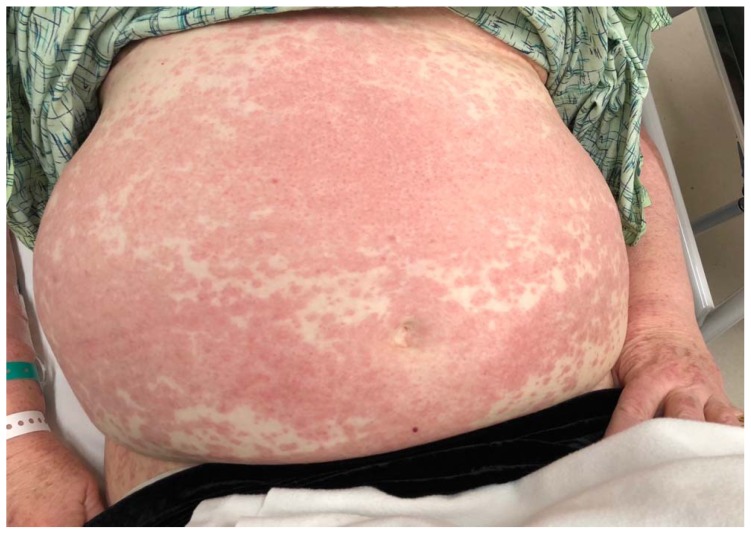
Abdominal rash.

**Figure 3:**
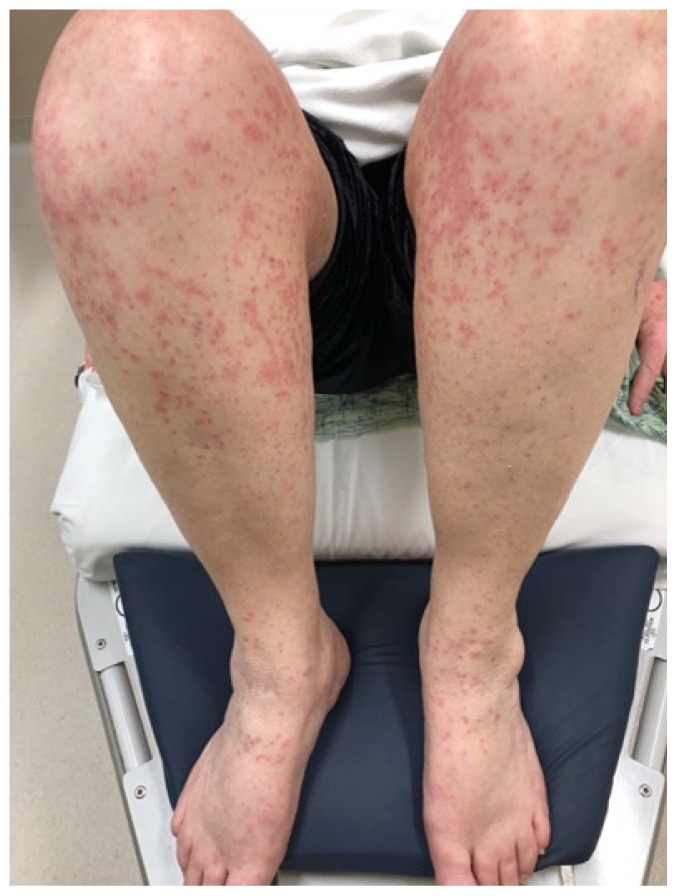
Rash of the leg.

**Figure 4:**
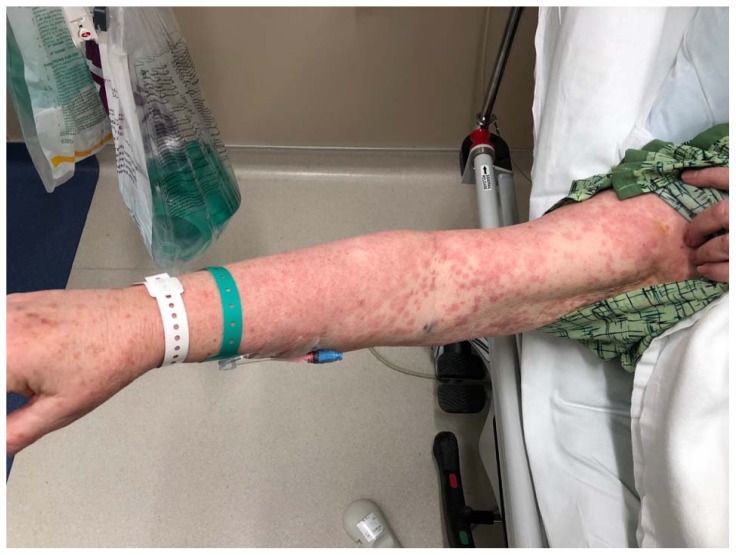
Rash of the arm.

A 74-year-old woman with a history of hypertension and coronary artery disease presented to the ED with a diffuse, intensely pruritic maculopapular rash of four days’ duration (Figures 1 - 4). She visited her primary care physician three days prior to her ED visit, where she denied any medication changes, reporting only her longstanding use of hydrochlorothiazide, lisinopril, and clopidogrel, of which she had been on stable doses for a year. She also denied environmental or occupational exposures. Her primary care physician (PCP) prescribed a course of glucocorticoids, and when the rash continued to progress despite therapy, she was instructed to go to the ED. While the patient had denied new medications or exposures to her PCP, in the ED, it occurred to her that she had been exposed to an herbal supplement that she had not mentioned during her clinic visit. She recalled that the week previous to the development of her rash, she had experienced chills and cough, and had been diagnosed by a naturalist with influenza and advised to take an extract marketed as “LDM-100,” an extract of *Lomatium dissectum*, a plant used as an herbal remedy for viral illnesses. She had taken this extract for two days as directed (six drops orally five times daily) when the rash erupted and continued taking it after the rash was present, but had stopped taking the extract the day before presentation to the ED because her cough and fever had resolved.

In the ED, a complete blood count, complete metabolic panel and inflammatory markers were unremarkable. It was deduced the *Lomatium dissectum* was the likely source of the rash, the patient was discharged home, and she elected to discontinue steroids. Five days after presentation, her symptoms had completely resolved.

## Discussion

*Lomatium dissectum* is a plant native to western North America and colloquially known as fernleaf biscuitroot.^3^–^5^ It is commonly marketed as “LDM-100” and has gained popularity among practitioners of herbal medicine as a treatment against influenza. As evidence of its effectiveness, herbalists point to anecdotal experience of its use against influenza, its *in vitro* activity against other viruses such as rotavirus, and the observation that Native American populations using *Lomatium* during the influenza pandemic of 1917–18 had low rates of infection.^6^,^7^ A side effect of the use of this plant that is known to the herbal medicine community is development of a pruritic, whole-body rash that appears within 1 – 3 days of initiating treatment with *Lomatium*, and generally resolves within 5–7 days of stopping exposure. Dosing regimens for *Lomatium* in the natural medicine community vary widely, from as few as 3 drops orally three times daily to 90 drops orally four times daily, with some sources suggest that lower initial doses that gradually increase are less likely to cause rash.^5^,^8^ Our patient’s experience was consistent with this, as she started at a relatively generous initial dose, and the rash disappeared within 5 days of cessation. In addition to the preparation used by our patient, *Lomatium* can also be ingested as the unprocessed plant, used in teas, and also prepared in “isolate” form with the resins removed, which is a form alleged in some sources (without clear evidence) to be less likely to cause rash. The naturopathic literature from which most information about rashes caused by *Lomatium* is drawn do not indicate a well-studied treatment of the rash aside from supportive care and cessation of *Lomatium* ingestion. In our patient, the rash was refractory to glucocorticoids and only resolved once her exposure to the extract ceased, although antihistamines were helpful to reduce pruritis. A strategy of withholding *Lomatium* and focusing on symptom relief would seem to be a reasonable approach in similar cases.

Prior to this case report, there were no reports of this reaction to *Lomatium dissectum* in the peer-reviewed medical literature. Considering the likely continued use of *Lomatium* for influenza and other viral illnesses and the dramatic nature of the rash, physicians should be aware of this side effect.

## References

[1] Uscher-Pines L, Elixhauser A (2013). Emergency Department Visits and Hospital Inpatient Stays for Seasonal and 2009 H1N1 Influenza, 2008–2009. HCUP Statistical Brief #147.

[2] Lovegrove MC, Shehab N, Hales CM, Poneleit K, Crane E, Budnitz DS (2011). Emergency department visits for antiviral adverse events during the 2009 H1N1 influenza pandemic. Public Health Rep.

[3] Moore M (1993). Medicinal Plants of the Pacific West.

[4] Thie K, Gladstar R, Hirsch P (2000). Lomatium. Planting the Future: Saving our Medicinal Herbs.

[5] Barlow J (2017). The Super Natural Power of Lomatium.

[6] Bergner P (2005). Antiviral botanicals in herbal medicine. Medical Herbalism.

[7] McCutcheon AR, Roberts TE, Gibbons E (1995). Antiviral screening of British Columbian medicinal plants**. J Ethnopharmacol.

[8] Stark A (2009). Lomatium Root: Possibly the Best Anti-Viral [Internet]. Debra’s Natural Gourmet.

